# Novel Multifunctional Spherosilicate-Based Coupling Agents for Improved Bond Strength and Quality in Restorative Dentistry

**DOI:** 10.3390/ma15103451

**Published:** 2022-05-11

**Authors:** Zbigniew Raszewski, Dariusz Brząkalski, Marek Jałbrzykowski, Daria Pakuła, Miłosz Frydrych, Robert E. Przekop

**Affiliations:** 1 R&D, SpofaDental, Markova 238, 506-01 Jicin, Czech Republic; zbigniew.raszewski@kerrdental.com; 2 Faculty of Chemistry, Adam Mickiewicz University in Poznan, 61-614 Poznan, Poland; dariusz.brzakalski@amu.edu.pl (D.B.); darpak@amu.edu.pl (D.P.); frydrych@amu.edu.pl (M.F.); 3 Faculty of Mechanical Engineering, Bialystok University of Technology, Wiejska 45 C, 15-351 Bialystok, Poland; m.jalbrzykowski@pb.edu.pl; 4 Centre for Advanced Technologies, Adam Mickiewicz University in Poznan, 61-614 Poznan, Poland

**Keywords:** adhesive, cage siloxane, light curable, oral medicine, POSS, SSQ, stomatology

## Abstract

The aim of this study was to investigate the restorative connections of composite materials after fracture, under controlled conditions of treating the materials with novel, spherosilicate-based (SS) primers bearing both methacryl (MA) and trimethoxysilyl (TMOS) groups. The chemistry of methacrylate group insertion and reactive groups hydrolysis has been studied with the aid of ^1^H NMR (Nuclear Magnetic Resonance) spectroscopy. The light-cured resin composites were repaired by activating the connection site with the obtained primers and, for comparison, a silane (methacryloxypropyltrimethoxysilane, MATMOS) as a conventional coupling agent bearing the same reactive groups. The resistance of such a joint was tested in a three-point bending test after 24 h and 28 days period of sample conditioning. The effect of bond application was also studied, showing that spherosilicate-based primers may be used more effectively than MATMOS for two-step (primer-composite) restorative process, while for silane, the three-step process with bond application is crucial for satisfactory joint quality. The joint failure mode was determined by microscopic analysis and it was found that SS-4MA-4TMOS and SS-2MA-6TMOS application resulted in mostly composite, and not joint, failure. After 28 days of conditioning, the flexural strength of the joint repaired with SS-4MA-4TMOS was at 94% of the neat, solid material under the same procedure. However, the strength of the neat composite was observed to decline during the conditioning process by ~30%. The joint behavior was explained on the basis of the gradual hydrolysis effect (the greatest decrease being observed for silane).

## 1. Introduction

Methacrylate-based composites are commonly used in various fields: adhesive, automotive industry, dentistry. This is because the combination of methacrylic resins with various types of fillers improves their mechanical properties, such as fracture resistance, shrinkage during polymerization, and hardness [1,2]. Nevertheless, during the use of such resistant materials, they may be damaged, especially when they are exposed to a very high compressive or shearing force in a wet or corrosive environment [3]. One such example is composites used in dentistry [4]. Therefore, instead of replacing them, there is an increasing need for improved methods of repairing these in restoration procedures. A primary step in preparation of the material for a restorative procedure is to remove the layer of material that has been altered by water adsorption with different types of diamond or carbide cutting tools or sandblasting with sharp edged alumina. As the result of using such tools is the creation of fresh, microrough surface of the composite material, to which new resin can be attached. For the newly created joint to have the highest mechanical resistance to breaking or bending, several mechanical and chemical procedures are used, the purpose of which is to increase the bond quality and bonding strength [5,6]. The second step is priming the prepared surface with a selected coupling agent, usually silane. In the third step, low viscosity methacrylic resins containing small amounts of filler, called bonds, are applied. The task of the low-viscosity material is to penetrate all the cavities of the repaired, microrough composite material [5]. Resins of this type contain low molecular weight methacrylates (ethylene glycol trimethacrylate-TEGDMA, or 2-hydroxyethyl methacrylate (HEMA)), which, according to some authors, may penetrate to some degree into the repaired composite material [7,8]. These types of bonding systems are usually polymerized with light or are light-assisted self-curing materials. In dentistry, however, due to their ease of use, light-polymerized materials are used [7]. After curing of the bond layer, a new layer of composite material is applied to the surface under repair and light-cured, and, if needed, reshaped with drills between applications, until the desired shape is obtained [5].

Silane coupling agents are commonly used as primers providing improved interaction between different surfaces. They may either impart increased wetting action of the polymer on the filler surface in polymer composites, increased blending of two polymers in polymer blends, or chemical bonding on the interface of the system components [9]. Nakonieczny et al. reported on the effective ZrO_2_ silanization with APTES (3-aminopropyltriethoxysilane) [10], while Mahdavi on silanization of ZnO with APTES and TPTMOS (3-thiopropyltrimethoxysilane) [11] and Li on Al_2_O_3_ silanization with APTES, AEAPTES (N-(2-aminoethyl)-3-aminopropyltriethoxysilane) and MATMOS [12]. Vacche et al. reported that application of APTES bearing protic aminofunctional group capable of interacting with polar fluoropolymer allowed for significant reduction of dielectric loss and increase of thermomechanical stability of BaTiO_3_/P(VDF-TrFE) composites [13]. Sabri discussed different coupling agents for ceramic fillers dedicated for dental composites and compared the effectiveness of silane-type and titanate-type coupling agents [14]. The important effect of primer overloading causing decline of the mechanical properties of the resulting composite, which was described in detail in Zanchi’s work [15]. For priming dental, methacrylate-based composites, the silanes used bear two reactive, orthogonal groups: a thiol, methacrylate, or vinyl group from one side of the molecule, and an alkoxy (methoxy or ethoxy) silyl group at the other end. The task of the latter is to create a bond with a filler based on glass or silica and zirconia, while the remaining ones react with methacrylate resin [16]. 

However, despite the use of these various complicated procedures and purpose-tailored material systems, the newly established connection often breaks, and the results of mechanical tests prove them to be mechanically inferior to the neat composite. This is due to the absorption of water through this junction and the gradual hydrolysis of the resin or primer components. The mechanism of silane hydrolysis has been described extensively in the literature [14,17,18]. In addition, other factors are considered, such as oversaturation of the joint with coupling agent, resulting in the joint interphase containing unreacted monomers, low molecular condensation products, or photocured phase much weaker than the bulk resin composite [15].

**Figure 1:**
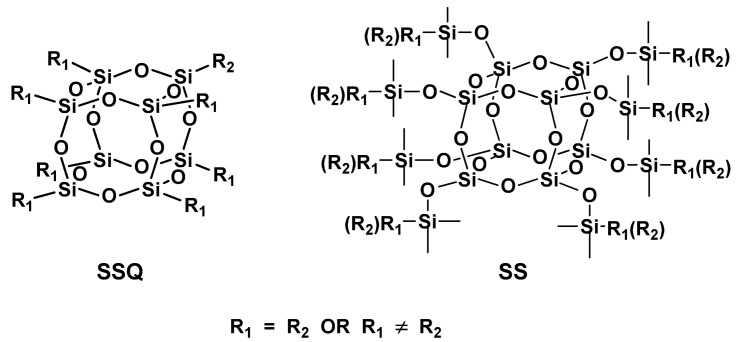
Structure of most common polyhedral oligomeric silsesquioxanes (SSQ) and spherosilicates (SS).

In recent years, however, other organosilicon molecules capable of coupling agent chemistry have been extensively studied—polyhedral oligomeric silsesquioxanes (POSS, SSQs), with their inorganic core made up of silicon and oxygen (SiO_1_._5_)_n_ [19]. Among silsesquioxanes, a subgroup known as spherosilicates (SS) is well established due to simplicity of their synthesis and catalytic modification (Figure 1). SS compounds have a characteristic structural feature of organosilyl M subunits protruding from the Si-O-Si cage, forming a functional organosilyl corona around the inorganic siloxane core. 

Both SSQ and SS compounds are spherical oligomeric species with multiple active centers, which can be modified with various substituents such as alkoxysilyl, methacryl, vinyl groups and many others, unreactive organic functional groups, which help SSQ molecules become soluble in, or compatible with, polymers [1,2]. Reactive groups enable SSQs and SS to serve as silane coupling agents for surface modification of inorganic fillers, as well as a reactive component of curable resins [20]. Adding such molecules to composite structure can greatly influence the physical properties, volumetric shrinkage, flexural strength, young modulus [21], biocompatibility and reduced inflammatory response [22].

Silsesquioxane molecules with various substituents showed higher miscibility with the dimethacrylate monomer. In the literature on methacrylate composites used in dentistry, these compounds have been successfully used as additives in a concentration of 1–50% [21,23,24,25], or as components of bonding systems [21,26,27]. However, these were materials where all active centers were substituted with the same reactive group: methacrylate. On the other hand, there are works where two kinds of substituents were present in the SSQ structure, however, the second group always being chemically inert, intrinsically bound to the silsesquioxane core chosen for the synthesis: ethyl group [28], isobutyl group [23,26]. Molecules with one reactive group can be used as anchoring points bound to the matrix chains and particles with many substituents can serve as cross-linkers, reducing the water uptake of the resins [23]. SSQ molecule containing Si-OR groups, which similarly as in the case of silanes, alleviated hydrolysis and can combine with the filler of the composite material. Methacrylic groups, as a result of free radical polymerization, are attached to the polymer chains [16]. However, the authors of this work did not manage to find in the literature information on the use of silsesquioxane or spherosilicate molecules containing different substituents (methacrylic and SiOR) groups in one molecule, providing both actions at the same time, studied in dental community.

The aim of this work was to test this type of hybrid, bifunctional compounds for improving of the connection between two composite materials under conditions of restorative dentistry procedure. The hypothesis put forward before the study was that SS compounds providing both reactive binding to the filler and to the methacrylate resin at the same time would increase the strength of the connection between composite materials and reduce the negative effects of strength decline over time during material conditioning. This is due to a higher number of reactive species per one molecule, and higher molecular mass, which both provide increased bonding action and create less water-soluble species prone to hydrolytic leaching under conditions of the composite service after restorative procedure. Additionally, this work discusses the effect of primer overloading on the surface of the primed surface and the importance of its careful application to obtained high quality of the repaired joint.

## 2. Materials and Methods

### 2.1. Materials and Instrumentation

The chemicals were purchased from the following sources: Tetraethoxysilane (TEOS), chlorodimethylsilane, tetramethylammonium hydroxide (TMAH) 25% methanol solution from ABCR (Karlsruhe, Germany), allyl methacrylate, methacryloxypropyltrimethoxysilane, toluene, chloroform-d, Karstedt’s catalyst xylene solution and isopropanol from Aldrich (St. Louis, MO, USA), P_2_O_5_ from Avantor Performance Materials Poland S.A. (Gliwice, Poland). Toluene was degassed and dried by distilling it from P_2_O_5_ under argon atmosphere. To test the connection strength of the joint, the composite materials Harmonize color Enamel XXL and Harmonize color A3.5, and a bond system Optibond Solo were obtained from Kerr Dental, Orange, CA, USA. The material was polymerized with a diode lamp emitting light in the 420 nm range (DemiUltra, Kerr Dental, Orange, CA, USA). Digital Light Microscope Keyence VHX 7000 with 100× to 1000× VH-Z100T lens and a VHX 7020 camera (Osaka, Japan). SEM-EDS analyses were recorded on a Quanta FEG 250 (FEI) instrument at 30 kV. ^1^H and COSY Nuclear Magnetic Resonance (NMR) spectra were recorded at 25 °C on a Bruker Ultra Shield 300 spectrometer using CDCl_3_ as a solvent. Chemical shifts are reported in ppm with reference to the residual solvent (CHCl_3_) peak.

### 2.2. Synthesis of Spherosilicate Compounds

Spherosilicate compounds were prepared according to literature reports: octahydrospherosilicate [29]; mixed group spherosilicates [20].

**Figure 2:**
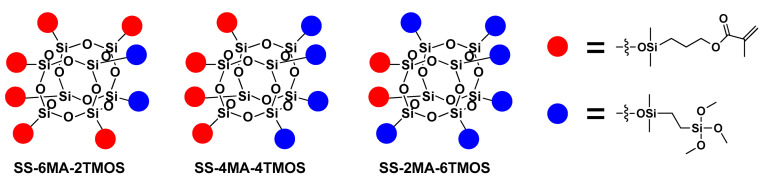
Idealized structures of the spherosilicate compounds used in this work.

The synthesized SS compounds were as follows: SS-6MA-2TMOS, SS-4MA-4TMOS, SS-2MA-6TMOS, where MA is an abbreviation for methacrylate group and TMOS for trimethoxysilyl group (Si(OMe)_3_), and the number next to the abbreviation gives the molar ratio of these groups introduced into the compound. The idealized structures of these compounds are given in Figure 2. It was found previously that for mixed groups SS compounds synthesis carried out by hydrosilylation route, a series of congeners is in fact generated, with the desired structure being the predominant, but not exclusive one, due to the normal distribution of the possible products [20]. Additionally, for the detailed NMR study purposes, SS-8MA derivative was synthesized under the same conditions as a spectroscopic standard for the study, to assign all the observed signals and resolve the structure of all the main and side products formed during allyl methacrylate hydrosilylation.

### 2.3. Samples Preparation

The obtained SS compounds were dissolved in isopropanol to a *w*/*w* concentration of 10% and the pH was lowered with 2 mL concentrated acetic acid (Sigma Aldrich, Prague, Czech Republic). For the tests, the solutions stored in this way were diluted with isopropanol to a concentration of either 2% or 0.1%, accordingly to the performed test. Dissolving SS in alcohol allowed for obtaining low-viscosity solution for ease of work, and, more importantly, for providing thin coating of the agent on the surface being promoted prior to joint repair, in order not to overload the surface with the agent.

As a comparative silane coupling agent, methacryloxypropyltrimethoxysilane in a 2% *w*/*w* isopropanol solution was applied with 2 mL of concentrated acetic acid, which is widely used in dentistry for this application.

To test the connection strength of the joint, samples of dimensions 2 mm × 2 mm × 25 mm were made from Harmonize color Enamel XXL by molding into metal molds and polymerizing with a diode lamp. The exposure time of the material to the light source was 20 s on each side. After removing the sample from the mold, it was broken (three-point) with a Shimadzu 450 compressive strength instrument (Tokyo, Japan). The test was carried out in accordance with the ISO standard 4049 [30]. 

The broken surfaces of composites were carefully polished with sandpaper, grouped and samples of each group treated with an alcoholic solution containing a given type of SS compound or silane (Table 1). After 10 min, when the alcohol solution had evaporated, the samples were separated into 2 groups. The first one was placed back in the same metal forms. The missing part, 12.5 mm long, was added from a new portion of Harmonize color A3.5. Using a different color composite allowed for tracing the joint surface after the joint repair. The whole assembly was placed in the hydraulic press (Dentalpress, Prague, Czech Republic) for a period of 1 min under a pressure of 1000 kg, so that the new composite material could fuse with the broken one. After removal from this instrument, the material sample was cured again with a LED lamp for 20 s on each side (Table 1, entries 1–6).

**Figure 3:**
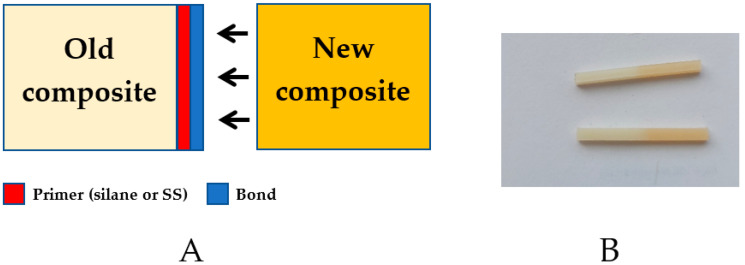
(**A**) Schematic reconstruction of the broken composite connection (**B**) photo of the repaired composite.

The bond was applied to the second group of samples, on the surface which was primed with solutions containing a selected SS or silane prior to repair. The material was polymerized for 40 s, according to the manufacturer’s instructions. After the bond curing, the samples were similarly placed in metal molds, and the missing parts were rebuilt with Harmonize color A3.5, placed in a press and polymerized (Table 1, entries 7–10). The process is presented on Figure 3.

To analyze the effect of the primer overloading and leftover primer monomers compromising continuity of the repaired joint, a test of monomer removal was performed. For this purpose, 2% SS or MATMOS solution was applied to the broken surfaces of the composite material, which, after drying for 30 min at room temperature, was subjected to solvent treatment in isopropanol. The process took 5 min in a 50 W ultrasonic cleaner. After repeated drying, the bonding system was applied to the surface, polymerized and then the composite material was applied in the fashion described above. (Table 1 entries 11–13). An alternative procedure was tested, not requiring the sonication of the samples, by simply diluting the primer solutions further to 0.1%, which ensured very low lading of the primers on the repaired surfaces (Table 1, entries 14–16).

### 2.4. Samples Conditioning and Flexural Strength Measurements

The samples were placed in distilled water in a laboratory oven at a temperature of 37 °C. Samples were measured either after 24 h or 28 days, by fracturing them in a three-point bending test on a Shimadzu 450 instrument (Shimadzu Europa GmbH, Duisburg, Germany), with breaking speed 0.5 mm/min. Mean fracture resistance and maximum deflection at the moment of sample breakage was detected. The remaining 6 samples from each group were stored for 28 days in distillate water at 37 °C at laboratory oven. The water was replaced once a week with new portions. After this period of time, the samples were broken.

## 3. Results and Discussion

### 3.1. Nuclear Magnetic Resonance

The NMR study of the post-reaction mixture of SS-H and allyl methacrylate (AM) revealed that the addition of Si-H to the unsaturated bonds of the olefin resulted in formation of a complex mixture of products. The reaction selectivity was calculated on the basis of ^1^H NMR and the signals were assigned with the aid of a COSY experiment. The main product observed was, as expected, an anti-Markovnikov product of Si-H addition to the allyl moiety of allyl methacrylate, which is common for olefin hydrosilylation (Table 2, product 1) [31]. 

**Figure 4:**
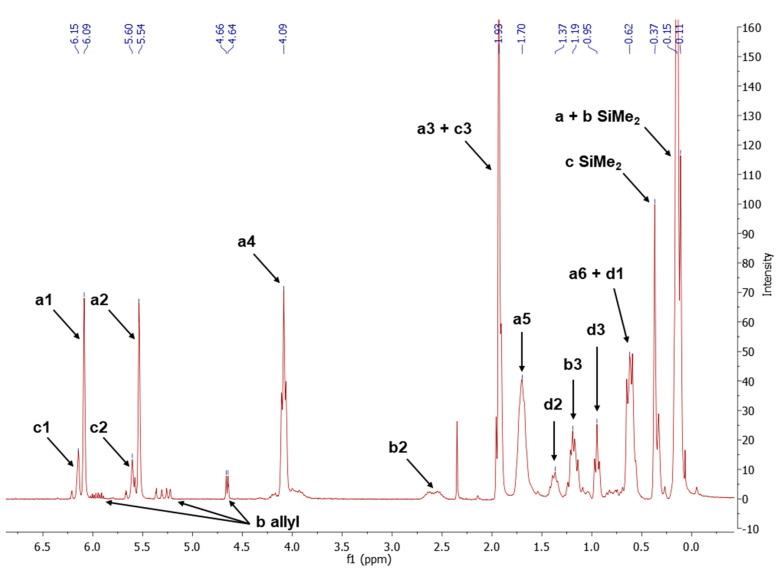
^1^H NMR spectrum of post-reaction mixture of allyl methacrylate hydrosilylation with SS-H.

The Markovnikov product formation was not confirmed, interestingly, although it might be formed in small amounts not detected due to the signals overlapping. Products 2 and 3 are the result of methacrylate moiety hydrosilylation, product 3 with the free allyl group remaining, while product 2 being a cross-linking group due to Si-H addition to the both carbon-carbon double bonds in the AM molecule. The products 4 and 5 are the result of back-biting reaction, common for allylic olefins bearing electron-withdrawing substituents [32]. The migration of methacrylate group from propyl chain to silicon atom causes formation of product 4, and upon elimination of propene molecule, its hydrosilylation results in formation of product 5. The resonance signals of characteristic nuclei were marked on the ^1^H NMR spectrum in Figure 4 with the symbols used in Table 2. The same products of AM hydrosilylation were observed when mixed group SS compounds (SS-6MA-2TMOS, SS-4MA-4TMOS, SS-2MA-6TMOS) were synthesized. It is suspected that formation of product 4 moieties in the post-reaction mixtures of spherosilicate derivatives used in this study results in increased ability of these molecules towards reaction with inorganic filler of the dental composite and formation of insoluble products less susceptible towards hydrolysis than silane primers. It is due to silanol generation by hydrolysis of labile methacryloxysilyl (acyloxysilyl) group with release of corresponding free methacrylic acid (MA) molecule.

**Figure 5:**
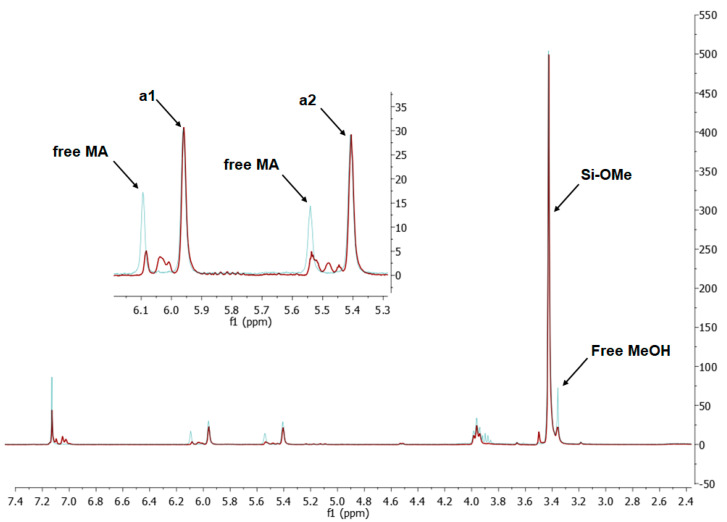
^1^H NMR spectrum of hydrolysis product of SS-4MA-4TMOS in iPrOH-H_2_O solution (blue) and neat SS-4MA-4TMOS (red). The spectra are superimposed for the comparison purpose.

The hydrolysis mechanism was confirmed by performing an experiment, where a sample of SS-4MA-4TMOS was dissolved in iPrOH (1 g/10 mL of iPrOH) and relatively small amount of water (3 mol eq/1 mol Si-OMe) was added, which allowed for producing gel for ^1^H NMR analysis by evaporating the sample to dryness. The obtained ^1^H NMR spectrum was then compared with the spectrum of neat SS-4MA-4TMOS. On the spectrum (Figure 5) there are resonance signals corresponding to free methacrylic acid from acyloxysilyl group hydrolysis, as well as free methanol signal from TMOS group hydrolysis. Small intensity of MeOH singlet is due to methanol loss during solvent evaporation. Si-OH (silanol) groups formed during hydrolysis are responsible for anchoring the SS molecule to inorganic filler of the dental composite, as well as formation of spherosilicate oligomers of higher molecular mass and lower solubility, which is discussed later.

### 3.2. Mechanical Analysis

**Figure 6:**
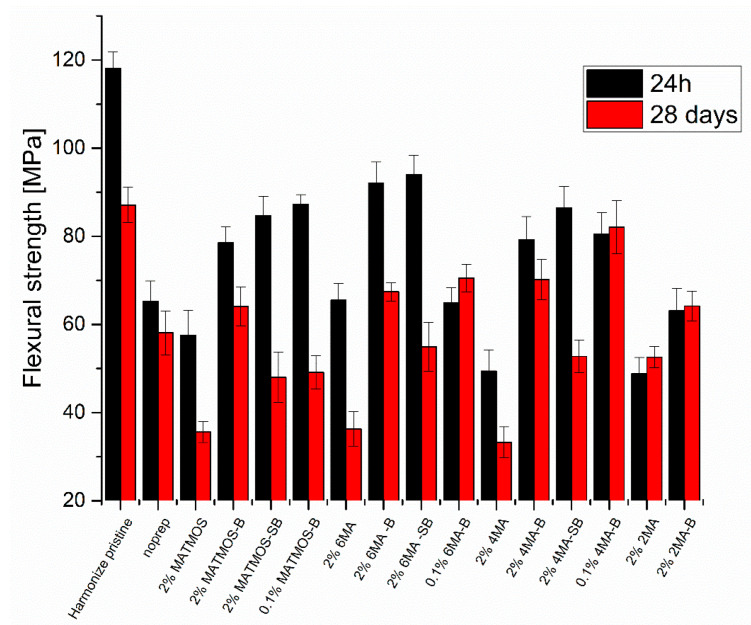
Flexural strength of composite materials before and after priming and repair.

**Figure 7:**
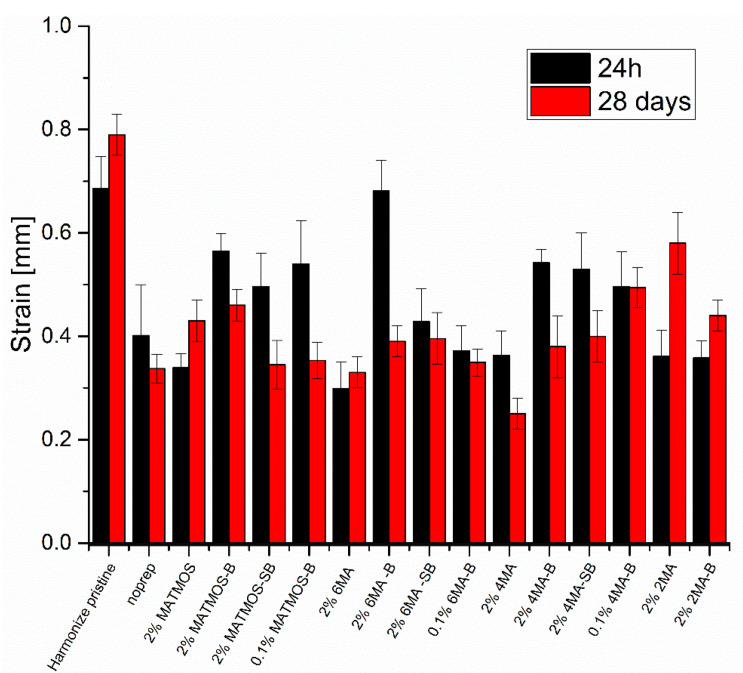
Strain of composite materials before and after priming and repair.

Results from the flexural tests after 24 h and 28 days are presented in Figure 6 and Figure 7, giving the flexural strength and strain, respectively. The fracture resistance of the composite material (Harmonize pristine) is 26% lower after 28 days of material curing. During storage in distilled water, this connection weakens due to gradual hydrolysis, until reaching plateau. On this basis, flexural stress of neat material after 28 days it was considered the base value for comparison with the repaired samples. The noprep sample batch (Table 1, Entry 2), made with no priming nor bond prior to composite application was prepared to present how poor such joint may perform mechanically. In this case, the drop of flexural strength over time is much lower, as the main source of sample failure is due to poor adhesion between materials, resulting in early fracture. In all cases, the importance of bond application is clearly visible, as a severe drop of fracture resistance in its absence. The application of sonication procedure, tested for MATMOS, SS-6MA-2TMOS and SS-4MA-4TMOS, resulted in small increase of initial flexural strength, however, the joint underwent failure under lower load after 28 days of curing, proving more severe hydrolytic degradation of the joint. In all cases, using 0.1% of the primer instead of 2% solution resulted in increased flexural strength after 28 days. This observation leads to the conclusion that overapplication of the primer is easily possible, leading to higher probability of the joint failure over time, and the primer should be of optimal concentration, or the excess thereof should be removed after application. This finding is in line of the Zanchi’s work [15]. On the other hand, sample sonication resulted in removal of too much primer from the surface prepared for repair. The most satisfactory results were observed for 0.1% of SS-4MA-4TMOS used with bond, where the joint strength after 24 h and 28 days was identical within SD levels, and the mean flexural strength after 28 days was at 94% of that of the neat composite, providing almost complete fusion of the joint materials. 

Additionally, 2% of SS-2MA-6TMOS used without bond was the most effective among all systems tested without bond application, hinting a possibility of further optimization of its application or development of more effective derivatives for two-step application with no need for bond utilization. However, the results prove that at present time, the use of bond is crucial for high joint performance and that SS-4MA-4TMOS system is preferred. These results are also supported by the analysis of strain at fracture (Figure 7). Application of bond increases this parameter in almost all cases, and among samples with no bond used, 2% SS-2MA-6TMOS system performed the best. Freshly repaired join utilizing 2% SS-6MA-2TMOS system with bond showed strain at fracture virtually identical to that of the pristine material, however the parameter value dropped significantly after 28 days due to hydrolysis.

### 3.3. Microscopic Analysis

**Figure 8:**
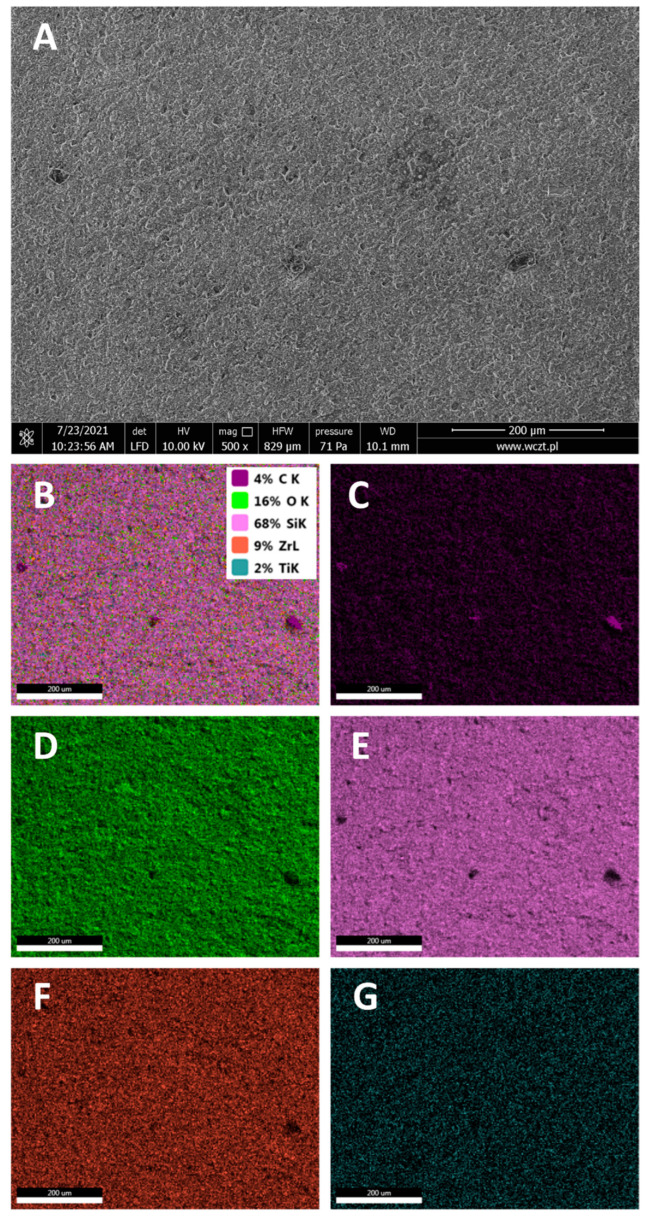
SEM (**A**) and EDS (**B**–**G**) images of the broken surface of neat composite. EDS maps show the presence of (**B**)—all detected elements in wt%, (**C**)—carbon; (**D**)—oxygen; (**E**)—silicon; (**F**)—zirconium; (**G**)—titanium.

Figure 8 presents the SEM image of a fractured surface of the neat Harmonize composite material. Silicon presence comes from microsilica filler; zirconium from zirconia used mostly as an RTG contrasting agent; titanium from TiO_2_ pigment used to balance the composite color; all these elements are in form of oxides, which is visible on oxygen EDS map. The oxides contain hydroxyl surface groups, which allow for chemical reaction with a silane or spherosilicate-based coupling agent (primer) [20].

**Figure 9:**
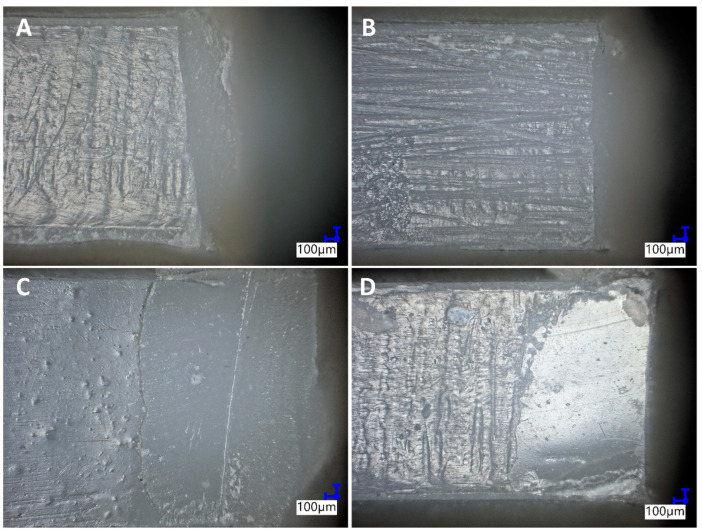
Digital optical micrographs of the studied systems: (**A**)—2% MATMOS; (**B**)—2% 6MA; (**C**)—2% 4MA; (**D**)—2% 2MA. For (**A**,**B**), joint adhesion failure was observed. For (**C**,**D**), mostly composite failure was observed.

Figure 9 represents the joints repaired with the studied systems (the batch without bond application), and fractured for the second time. Due to application of two composite colors (Harmonize Enamel and Harmonize A3.5), it is clearly visible if the adhesion failure occurred at the adhesion site or out of adhesion site. MATMOS and 6-MA systems at 2% concentration showed a tendency towards adhesion failure, while for 4-MA and 2-MA most of the samples of the studied systems proved to fail near the adhesion site, but often in a mixed mode, where some joint failure is visible together with composite failure. When compared with the sample batch with the bond applied, most of the samples proved to fail in a mixed mode, with most of the fracture cross-section being composite that fractured outside of the joint surface. This confirms that an adhesive type of connection was observed for the studied systems, which is consistent with the study of other authors [33,34,35].

As noted by Li [36] (a pioneer in this field) three mechanisms influence the connection of the repaired composite: (1) micromechanical bonding due to surface irregularities, (2) chemical bonding between two resin matrices and (3) chemical bonding with the filler. For this study, the mechanical method of surface preparation described above provided the surface irregularities, while different coupling agents were proven to generate an interphase with different levels of composite compatibilization and hydrolytic stability. The newly formed joint is weaker than those before fracture, which is in line with the work of Eliasson et al. [37]. Additionally, the authors noted that not curing the bond prior to applying the composite material improves the bond strength. They believe that the thinner the bonding layer, the greater the bond strength. 

The chemical bond between the resin matrix in the two composites can be achieved with spherosilicate molecules bearing multiple methacrylate groups reacting with either bond or fresh composite, and alkoxysilyl groups reacting with surface hydroxyl groups of the inorganic fillers (silica and zirconia) of both old and fresh composites (or fillers of old composite and bond). Changing the ratio of MA and TMOS groups allowed for finding that SS-6MA-2TMOS system was the poorest performing of the tested spherosilicate primers, probably due to too high methacrylate content, causing self-curing instead of reacting with the bond or fresh composite, and giving too little anchoring of the primer to the primed surface. This, in turn, resulted in the subsequent hydrolytic elimination of the primer from the joint over the time of sample conditioning. Similarly, MATMOS, as a low-molecular agent, may undergo poor condensation under the studied conditions, resulting in its condensation products being hydrolytically eliminated from the joint during conditioning. Spherosilicate molecules of proper reactive group ratio may solve this problem by being less prone to hydrolysis, as suggested by the results obtained with SS-4MA-4TMOS and SS-2MA-6TMOS. 

## 4. Conclusions

A series of mixed substituent-bearing spherosilicate derivatives was synthesized, studied by the means of ^1^H NMR spectroscopy and tested as a novel class of primers for restorative dentistry of methacrylic resin composites. The effect of composite strength decline over time was discussed and the results were in line with the findings from other reports describing the effect of hydrolysis of the composite components. The most important conclusions from this research are as follows:

(1) The spherosilicate-based primers varied in efficacy and the best results were obtained for SS-4MA-4TMOS applied together with the bond, but the tests revealed that with proper substituents proportion and primer application, it might be possible to omit the bond application with this class of compounds, which was the most visible for SS-2MA-6TMOS.
(2) Using primer in lower concentration (0.1%) resulted in increased flexural strength after 28 days. This observation leads to the conclusion that overapplication of the primer is easily possible, leading to higher probability of the joint failure over time, and the primer should be of optimal concentration, or the excess thereof should be removed after application.
(3) Trials with eliminating the excess primer by sonication caused removal of too big fraction of it.
(4) The tests with SS-4MA-4TMOS and SS-2MA-6TMOS revealed the tendency of the composite to undergo failure of the composite instead of the joint, and that after 28 days of conditioning, the flexural strength of the joint repaired with SS-4MA-4TMOS with bond application was at 94% of the neat, solid material under the same procedure.

The obtained results are the starting point for further investigation of such systems, especially a detailed study covering the optimization of their application.

## Figures and Tables

**Table 1:** Samples prepared for tests.

Entry	Sample Description	Bond	Sample Code
1	HE ^1^	N/A	Harmonize pristine
2	HE/Harm. A3.5 without surface preparation	-	noprep
3	HE/2% SS-6MA-2TMOS/Harm. A3.5	-	2% 6MA
4	HE/2% SS-4MA-4TMOS/Harm. A 3.5	-	2% 4MA
5	HE/2% SS-2MA-6TMOS/Harm. A 3.5	-	2% 2MA
6	HE/2% MATMOS/Harm. A 3.5	-	2% MATMOS
7	HE/2% SS-6MA-2TMOS/bond/Harm. A3.5	+	2% 6MA-B
8	HE/2% SS-4MA-4TMOS/bond/Harm. A3.5	+	2% 4MA-B
9	HE/2% SS-2MA-6TMOS/bond/Harm. A3.5	+	2% 2MA-B
10	HE/2% MATMOS/bond/Harm. A3.5	+	2% MATMOS-B
11	HE/2% SS-6MA-2TMOS/sonication/bond/Harm. A3.5	+	2% 6MA-SB
12	HE/2% SS-4MA-4TMOS/sonication/bond/Harm. A 3.5	+	2% 4MA-SB
13	HE/2% MATMOS/sonication/bond/Harm. A 3.5	+	2% MATMOS-SB
14	HE/0.1% SS-6MA-2TMOS/bond/Harm. A3.5	+	0.1% 6MA-B
15	HE/0.1% SS-4MA-4TMOS/bond/Harm. A3.5	+	0.1% 4MA-B
16	HE/0.1% MATMOS/bond/Harm. A3.5	+	0.1% MATMOS-B

^1^ HE—Harmonize Enamel.

**Table 2:** Chemo- and regioselectivity of the allyl methacrylate hydrosilylation with SS-H.

Product	Product Structure ^1^	R Substituent	Content (mol%)
1	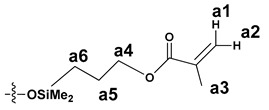	-	55
2	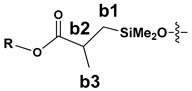	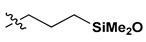	12
3	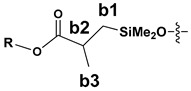	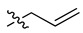	3
4	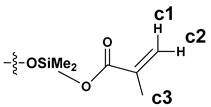	-	17
5	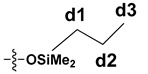	-	10

^1^ For all the structures given, OSiMe_2_ group is attached directly to the spherosilicate core, as shown in Figure 2.
